# Dark seasons enhance brain and brown adipose tissue interactions related to mu-opioid receptor signaling

**DOI:** 10.1007/s00259-025-07272-5

**Published:** 2025-04-17

**Authors:** Lihua Sun, Anne M. Landau, Jing Tang, Anne Roivainen

**Affiliations:** 1 Huashan Institute of Medicine, Huashan Hospital, Fudan University, Shanghai, 200031 China; 2 Turku PET Centre, University of Turku, Turku, FI-20520 Finland; 3 Turku PET Centre, Turku University Hospital, Turku, FI-20520 Finland; 4 Translational Neuropsychiatry Unit, Department of Clinical Medicine, Aarhus University, Aarhus N, 8200 Denmark; 5 Research Program in Systems Oncology, Faculty of Medicine, University of Helsinki, Helsinki, FI-00014 Finland; 6 Turku Center for Disease Modelling, University of Turku, Turku, FI-20520 Finland; 7 InFLAMES Research Flagship Center, University of Turku, Turku, FI-20520 Finland

**Keywords:** Brown adipose tissue, Mu-opioid receptor, Photoperiod, Brain-body interaction, Positron emission tomography

## Abstract

**Purpose:**

Prior studies reveal seasonal variations of mu-opioid receptor (MOR) signaling in both the brain and the brown adipose tissue (BAT). However, the potential seasonality effect on brain-BAT interactions, related to this signaling pathway, remains unknown. Understanding this dynamic seasonal rhythm may provide novel insights into seasonal affective changes and related psychiatric disorders.

**Methods:**

Nine adult rats (6 males and 3 females) were housed under standard conditions with photoperiodic cycles simulating local seasonal changes. The rats underwent repeated [^11^C]carfentanil PET imaging to assess MOR availability in the brain and BAT. Partial Least Squares Regression (PLSR) analysis was applied to evaluate the predictability of brain MOR availability on corresponding BAT measures. Latent variables in the PLSR models were eventually categorized by photoperiod.

**Results:**

PLSR models indicated that brain MOR availability considerably accounted for the variance of MOR levels in the BAT (22.82%), comparable to age (23%). Models applying different brain regional measures (striatum, neocortex and thalamus) produced consistent latent variables across models. A shorter photoperiod was associated with increased latent variable (beta = -4.32, 95% CI [-5.30, -3.35]).

**Conclusion:**

These findings suggest that shorter photoperiods enhance, while longer photoperiods reduce, the predictability of brain MOR levels on BAT MOR signaling. These data imply that darker seasons may amplify the interaction between brain activity and peripheral physiology associated with MOR signaling. The adaptability of brain-BAT interactions under stress stimuli offers a new avenue for exploring systems biology.

## Introduction

Recent studies highlight seasonal variations of mu-opioid receptor (MOR) signaling in both brain [1] and the peripheral brown adipose tissues (BAT) [2]. Endogenous opioid signaling, primarily via MORs, plays crucial roles in pain regulation [3], reward [4], social emotions [5], and inflammation responses [6]. The biological rhythms of this receptor signaling are closely linked to social well-being, yet our current understanding of these aspects remains incomplete. Prior studies focus on MOR availability at the individual-organ level, but cross-organ communication [7, 8] could provide a more comprehensive picture of the MOR system. A deeper understanding of seasonal variation in the MOR system may offer new insights into seasonal affective disorders (SADs).

Building on our previous findings [1, 2], we investigated whether brain-BAT communication varies with photoperiodic cycles. The brain-BAT interaction may occur via heat-mediated metabolic crosstalk [9], as well as endocrine and neuronal signaling [8]. Expression of MORs is found in both neurons and adipocytes [2], suggesting a potential role for endogenous opioids as messengers in cross-organ communications. Here, we propose that the MOR-associated brain-BAT communication is either enhanced or diminished during darker seasons, which are often associated with the onset of SADs.

## Methods

### Data

**Fig. 1:**
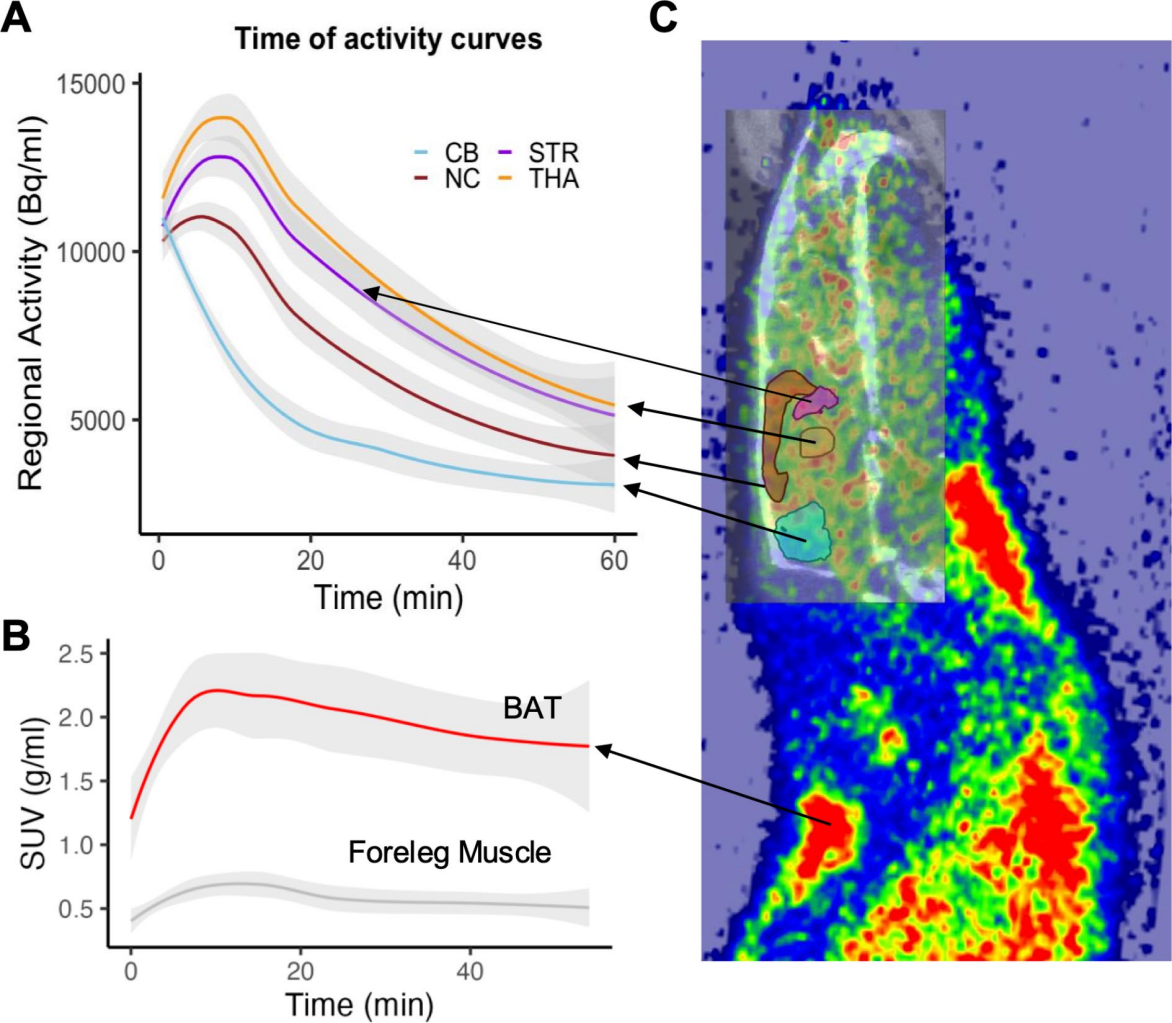
[^11^C]carfentanil PET imaging of mu-opioid receptor availability in rats. **(A)** Mean time–activity curves for regional activities in the brain, with shaded area for 95% confidence interval (CI). CB = cerebellum, STR = striatum, NC = neocortex, THA = thalamus. **(B)** Mean standardized update values (SUV) in brown adipose tissue (BAT) and foreleg muscle. **(C)** Overlaid PET and CT images of a rat. The PET image shows average signals across the entire scan period. The figure is modified from previous studies [1, 2]

Data on brain and BAT MOR availabilities, as previously described in our original studies [1, 2], were reanalyzed to investigate the dynamic brain-BAT interactions under photoperiodic cycling. Nine (6 male and 3 female) rats underwent repeated [^11^C]carfentanil PET measurements across 3 to 4 sessions, under photoperiodic cycles designed to simulate local daylength variations (30 scans in total). Each PET scan measured MOR availability in the brain (Fig. 1A&C) and BAT (Fig. 1B&C).

Briefly, rats were scanned using 60-min dynamic [^11^C]carfentanil PET under isoflurane anesthesia, with a 5 MBq dose (actual 4.69 ± 0.60 MBq), corresponding to 29.28 ± 20.53 ng/kg. An Inveon Multimodality PET/CT scanner (Siemens Medical Solutions, Knoxville, TN, USA) was used to image the male rats (two rats at a time), whereas a Molecubes PET/CT scanner (Gent, Belgium) was used for the female rats. Dynamic PET images were analyzed using Carimas software (version 2.10.3.0), developed at the Turku PET Centre, Finland. Brain regional MOR availabilities were estimated using a simple reference model that provides non-displaceable binding potential. In BAT, MOR availability was expressed as the ratio between BAT and foreleg muscle binding, as muscle was found to be an ideal peripheral reference region with lower MOR expression.

### Statistical analysis

#### Partial Least Squares Regression

We used Partial Least Squares Regression (PLSR) analysis to examine how brain MOR availability predicts BAT MOR availability. PLSR was chosen for its effectiveness with complex, high-dimensional data, including collinear or noisy variables [10]. By extracting shared variance, PLSR highlights key links between brain and BAT MOR levels, clarifying peripheral-central nervous system interactions without overfitting. Brain regional values (component 1) and Age (component 2) were the predictors (X), and BAT values were the outcomes (Y). The pls package in R with 10-fold cross-validation was used, with both BAT and brain values log-transformed.

#### Regression analysis of PLSR latent variables

The PLSR latent variables (LVs) were analyzed separately for LV1 (for component 1) and LV2 (for component 2) using a mixed-effects model, with each rat as a random effect and the photoperiod as a fixed effect. The lme4 package was used.

#### Latent variable clustering analysis

The goal was to identify distinct subgroups based on the multivariate structure of the data and examine their functional relationship with photoperiod. We applied an unsupervised k-means clustering algorithm separately to LV1 and LV2. To determine the optimal number of clusters, we employed the *Elbow* Method, which evaluates the within-cluster sum of squares (WSS) across a range of cluster numbers (k). The point at which adding more clusters results in a diminishing reduction of WSS is considered the optimal k. We used the factoextra package in R to visualize the WSS curve. Based on these plots, we selected the optimal cluster numbers for LV1 and LV2 separately. K-means clustering was performed with the selected number of clusters using 25 random initializations to ensure the stability of the results. To assess how cluster membership varied with photoperiod, we plotted the clusters as a function of photoperiod using jittered scatter plots. Dotted horizontal lines were used to indicate the range of photoperiod values observed within each cluster. Separate plots were generated for LV1 and LV2 to highlight potential differences in clustering patterns.

## Results

**Fig. 2:**
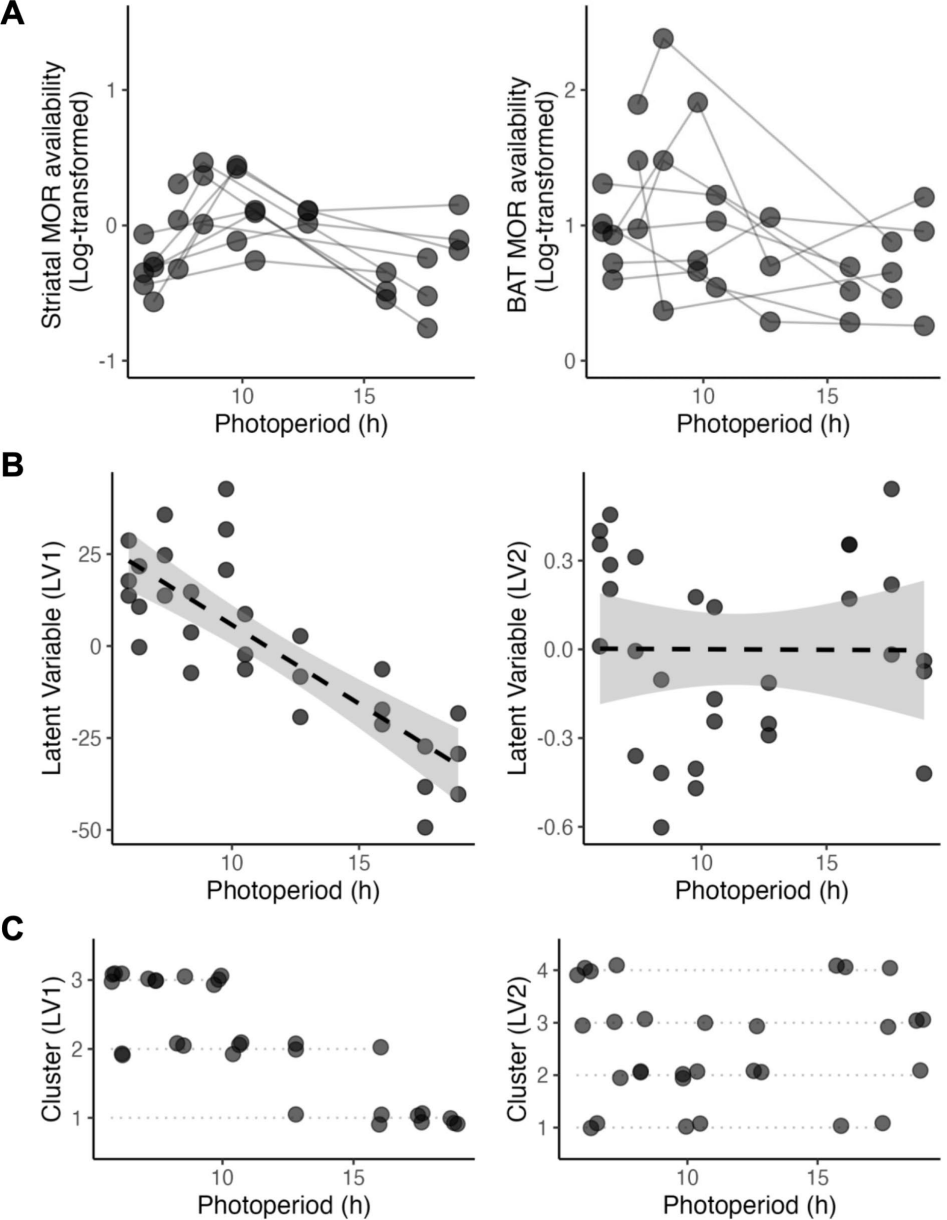
The predictability of striatal mu-opioid receptor availability on corresponding brown adipose tissue measures as a function of photoperiod. (**A**) Distribution of photoperiod in the striatal and BAT data. Data points from the same rat were connected by lines. (**B**) Relationship between latent variables and photoperiod. The shaded area represents 95% CI in both plots. (**C**) Cluster analysis of latent variables LV1 (striatal MOR availability) and LV2 (Age), regarding the relationship with photoperiod. BAT = brown adipose tissue; MOR = mu-opioid receptor, h = hour

MOR availability in BAT and brain (as exemplified by striatal values) are shown in Fig. 2A.

### PLSR analysis results

PLSR analysis showed that brain regional MOR availability considerably explained variance of MOR levels in the BAT (Table 1), comparable to Age. The three models generated consistent latent variables when “component 1” represents different brain regional values.

**Table 1:** Results of the Partial Least Squares Regression analysis

Brain Regions	RMSEP (Comp 1)	RMSEP (Comp 2)	Variance Explained
Striatum	0.4747	0.4905	22.82% (Comp 1); 23.00% (Comp 2)
Neocortex	0.4597	0.4734	22.82% (Comp 1); 23.37% (Comp 2)
Thalamus	0.4534	0.4708	22.82% (Comp 1), 23.09% (Comp 2)

N.B. Comp 1 is brain regional value and Comp 2 is Age; Comp = component, RMSEP = Root mean squared error of prediction

### Regression analysis of *latent variables*

Analysis of LVs indicated that shorter photoperiods were associated with increased LV1 units (Table 2). Higher latent variables suggest stronger covariation between brain and BAT MOR availabilities. LV2 was not affected by photoperiod.

**Table 2:** Regression analysis of latent variables as a function of photoperiod

Brain Regions	Latent Variables (LV)	Beta	95% CI	*p*-value
Striatum	LV1	-4.3253	[-5.30, -3.35]	< 0.001
	LV2	-0.0004	[-0.03, 0.03]	0.975
Neocortex	LV1	-4.3253	[-5.30, -3.35]	< 0.001
	LV2	-0.0062	[-0.04, 0.03]	0.738
Thalamus	LV1	-4.3254	[-5.30, -3.35]	< 0.001
	LV2	0.0017	[-0.02, 0.03]	0.890

N.B. LV1 refers to different brain regions in different models, while LV2 refers to Age

The functional relationship between LVs, when striatal MOR availability was “component 1”, and photoperiod is illustrated in Fig. 2B.

### Cluster analysis of the Latent Variables (LV)

The *Elbow* Method revealed that LV1 exhibited three optimal clusters, while LV2 exhibited four clusters (Fig. 2C). K-means clustering grouped scans based on their latent variable scores, revealing distinct mean values in both peripheral and brain measures. Clusters showed systematic variation with photoperiod, where the LV1 clusters displayed a relatively gradual transition across increasing photoperiod, while LV2 clusters exhibited a more nonlinear pattern (Fig. 2C). Results further highlight the impact of photoperiod on LV1.

## Discussion

Our key finding is that short days promote the communication between brain and BAT MOR signaling, as shown by increased predictability of brain regional MOR availabilities on the corresponding BAT measures. The increased crosstalk between the brain and BAT in dark seasons may suggest a whole-body-level adaption to seasonal photoperiod changes. Enhanced innervation from the brain to BAT, or the feedback signal from BAT to the brain, may be crucial in tuning energy homeostasis, social behavior, and immunity.

Peripheral measures often show larger measure-to-measure variabilities, as compared to brain measures. This is possibly caused by the blood-brain barrier, which buffers radical changes of multiple physiological factors and restricts the mobility of radiotracers and metabolites of the tracers. Similar latent variables were yielded when using different brain regional measures to predict BAT measures, and this may be explained by the larger between-animal variances of BAT values. Traditional correlation analysis may not be always feasible in linking the highly variable peripheral measures with brain correspondents [8], especially considering the continuous photoperiodic changes. Instead, the current study maximizes this predictability by assigning latent variables where higher latent variables suggest higher predictive power. Interestingly, these predictive powers are explained by photoperiod on the day of PET scanning.

Brain MOR availability exhibits an inverted-U functional relationship with increasing photoperiod, while BAT MOR signaling shows a continuous decline as days grow longer. This interplay naturally complicates their potential associations. Our finding that the predictability of brain MOR levels on corresponding BAT measures follows an ideal linear relationship with photoperiod and suggests a more complex domain of interactions beyond simple covariations. Importantly, this interaction is intricately linked with the external photoperiodic stimuli.

### Limitations

We have a relatively small number of points despite the significant findings. Based on the current analysis, the causal relationships between BAT and brain MOR signaling cannot be established. Also, only a linear relationship between BAT and brain MOR availability was investigated, leaving the possibly more complicated interactions unspecified.

## Conclusions

Our data show that dark seasons enhance brain-BAT interactions related to MOR signaling. This finding suggests the need for future studies to inspect the seasonal effects from the perspective of body-brain communications. The observed seasonal patterns of brain-BAT interactions may also apply to other stimuli, such as stress and pain, providing a novel framework for advancing systems biology.
